# High-Level Alzheimer Disease Neuropathological Change Following Iatrogenic Exposure

**DOI:** 10.1001/jamaneurol.2026.0437

**Published:** 2026-03-30

**Authors:** Gargi Banerjee, Tze How Mok, Harpreet Hyare, Oliver Cousins, Zane Jaunmuktane, Simon Mead, John Collinge

**Affiliations:** 1 MRC Prion Unit at UCL and UCL Institute of Prion Diseases, London, United Kingdom; 2 National Prion Clinic, National Hospital for Neurology and Neurosurgery, London, United Kingdom; 3 UCL Queen Square Institute of Neurology, London, United Kingdom; 4 Lysholm Department of Neuroradiology, National Hospital for Neurology and Neurosurgery, London, United Kingdom; 5 St George’s University Hospitals NHS Foundation Trust, London, United Kingdom; 6 Department of Clinical and Movement Neurosciences and Queen Square Brain Bank for Neurological Disorders, UCL Queen Square Institute of Neurology, London, United Kingdom; 7 Division of Neuropathology, National Hospital for Neurology and Neurosurgery, London, United Kingdom

## Abstract

**Importance:**

Alzheimer disease (AD) is pathologically characterized by the deposition of amyloid-β (Aβ) and hyperphosphorylated tau. Human transmission of Aβ pathology in a prion-like fashion has resulted in iatrogenic cerebral amyloid angiopathy. More recently, iatrogenic Alzheimer disease (iAD) was described in recipients of cadaveric pituitary–derived human growth hormone (c-hGH) contaminated with Aβ amyloid seeds.

**Objective:**

To describe the clinical and postmortem findings in iAD.

**Design, Setting, and Participants:**

This case series describes 4 c-hGH recipients who were referred to the UK National Prion Clinic. Between February 2024 and February 2025, 14 c-hGH recipients had been referred to this service, with clinical assessments ongoing. The current study included 4 of 14 people treated with c-hGH who were referred since the original report. These data were analyzed during February and March 2025.

**Exposure:**

c-hGH contaminated with Aβ amyloid seeds.

**Main Outcomes and Measures:**

Clinical and histopathological description.

**Results:**

The study describes 4 males who developed dementia following confirmed or suspected c-hGH treatment in childhood (age at symptom onset between 47 and 60 years) with cognitive syndromes characterized by prominent language involvement. Results include clinical and postmortem findings for 1 patient (onset at age 47 years) in whom postmortem examination (at age 57 years) showed unequivocal neuropathological features of AD, including severe tauopathy. Brief descriptions of 3 additional patients with prominent language involvement are also provided.

**Conclusions and Relevance:**

These results demonstrate that patients with iAD can have histopathological findings classically found in sporadic AD and that prominent language involvement might be an important phenotypic feature in this AD subtype.

## Key Points

**Question:**

What are the clinical and postmortem findings in iatrogenic Alzheimer disease (iAD) consequent to treatment with cadaveric pituitary–derived human growth hormone (c-hGH)?

**Findings:**

This case series describes a c-hGH recipient with early-onset dementia and prominent language involvement, in whom postmortem examination showed unequivocal neuropathological features of AD, including severe tauopathy. Three additional c-hGH recipients have similar cognitive syndromes characterized by prominent language involvement.

**Meaning:**

These results demonstrate that patients with iAD can have histopathological findings classically found in sporadic AD and that prominent language involvement might be an important phenotypic feature in this AD subtype.

## Teaser

This case series describes the clinical and postmortem findings in iatrogenic Alzheimer disease.

## Introduction

The expanded prion paradigm posits that self-propagating assemblies of misfolded proteins forming amyloid fibrils are crucial in the development of many neurodegenerative diseases, including Alzheimer disease (AD), Parkinson disease, amyotrophic lateral sclerosis, and several tauopathies.^1,2^ While there are substantial data from in vitro and in vivo model systems for the aggregating protein assemblies concerned, the prion protein (PrP) and amyloid-β (Aβ) remain unique as examples where human transmission of pathology has been established with resulting clinical disease. Several cases of iatrogenic cerebral amyloid angiopathy (iCAA) and parenchymal Aβ pathology have been reported internationally since our initial histopathological description in recipients of cadaveric pituitary–derived human growth hormone (c-hGH) who had developed iatrogenic Creutzfeldt-Jakob disease (iCJD).^3,4^ Patients with clinical iCAA typically present with intracerebral hemorrhage decades after the causative medical procedure, which generally involved cadaveric dura mater^5,6^; intracerebral hemorrhage risk also appears increased in recipients of cadaveric pituitary hormones.^7^ More recently, we described iatrogenic AD (iAD) in patients treated with c-hGH who had not succumbed to iCJD, again following a latent period of decades.^8^ Direct experimental evidence for Aβ transmissibility, long established in experimental animals, is obviously not possible in humans. However, the association between a particular c-hGH preparation known to contain Aβ seeding activity^4^ and early-onset AD in the absence of genetic risk factors or other plausible causation is compelling. Despite these data, others have questioned whether these cases meet the full diagnostic criteria for AD.^9^ We maintain that we are describing a novel condition, iAD, the full phenotypic range of which is yet to be described and which we expect to differ from both sporadic and inherited AD. This is the case in human prion diseases, where iCJD is neuropathologically similar to sporadic CJD but can have different clinical features in life.^10^ While reiterating this assertion, we accept that only limited neuropathology was available in our previous report and that this showed only mild tauopathy.^8^

Since the publication of our earlier report,^8^ the UK National Prion Clinic (NPC) has received multiple inquiries regarding individuals previously treated with c-hGH. Here, we provide a detailed clinical and postmortem description of one such recipient and briefly summarize other relevant referrals received to date.

## Methods

The NPC forms part of the United Kingdom national referral system for suspected prion diseases and coordinates the National Prion Monitoring Cohort,^11^ a longitudinal study of individuals with confirmed prion diseases (sporadic, inherited, iatrogenic, or variant forms) and those at risk of inherited, iatrogenic, or variant CJD, including people previously treated with c-hGH.

Data presented here were collected during the provision of routine clinical care and have been anonymized to prevent patient identification. Consent for publication of deidentified clinical data was provided by all patients and/or their next of kin. The National Prion Monitoring Cohort has been reviewed and approved by the following National Health Service Health Research Authority Research Ethics Committees: Scotland A Research Ethics Committee (reference: 05/MRE/0063); London Queen Square Research Ethics Committee (reference: 24/LO/0510). Additional approvals, where applicable, are provided in the sections below. Reporting is in accordance with EQUATOR (CARE) guidelines.^12^

### Data Availability

All available deidentified clinical data are included in this published article. Patient identifiable information, including genetic data, cannot be made publicly available for reasons of patient privacy and confidentiality but are available from the corresponding author upon reasonable request with supporting ethical approval.

### Genetic Testing

Written informed consent for genetic testing was obtained. Next-generation sequencing was performed commercially by Centogene, using methods described previously.^8^

Variants (including copy number variants) in the following genes associated with adult-onset neurodegeneration were reviewed: *ATXN2, ABCA7, ALS2, ANG, ANXA11, APOE, APP, ARSA, ATL1, ATP7B, BSCL2, C9orf72, CCNF, CHCHD10, CHMP2B, CP, CSF1R, CYLD, CYP27A1, DCTN1, ERBB4, EWSR1, FIG4, FTL, FUS, GLE1, GRN, HEXA, HNRNPA1, HNRNPA2B1, HSPD1, ITM2B, KIF5A, MAPT, MATR3, MT-ATP6, MT-ATP8, MT-CO1, MT-CO2, MT-CO3, MT-CYB, MT-ND1, MT-ND2, MT-ND3, MT-ND4, MT-ND4L, MT-ND5, MT-ND6, MT-RNR1, MT-RNR2, MT-TA, MT-TC, MT-TD, MT-TE, MT-TF, MT-TG, MT-TH, MT-TI, MT-TK, MT-TL1, MT-TL2, MT-TM, MT-TN, MT-TP, MT-TQ, MT-TR, MT-TS1, MT-TS2, MT-TT, MT-TV, MT-TW, MT-TY, NEFH, NEK1, NOTCH3, NPC1, OPTN, PANK2, PFN1, PRNP, PRPH, PSEN1, PSEN2, REEP1, SETX, SIGMAR1, SLC52A3, SNCA, SOD1, SORL1, SPAST, SPG11, SQSTM1, TAF15, TARDBP, TBK1, TFG, TREM2, TUBA4A, TYROBP, UBE3A, UBQLN2, VAPB, VCP, *and* WASHC5.*

### Postmortem Brain Tissue Preparation

Informed consent to use the tissue for research was obtained from the next of kin and ethical approval was obtained from the local research ethics committee of the University College London Queen Square Institute of Neurology. Brain-restricted autopsy was carried out in a postmortem room designated for high-risk autopsies. Postmortem tissues were extensively sampled from multiple brain regions. Tissue samples were immersed in 10% buffered formalin and potential prion infectivity was inactivated by immersion into 96% formic acid for 1 hour, followed by further fixation in formalin and processing to paraffin wax. Tissue sections were routinely stained with hematoxylin-eosin, followed by immunostaining with anti-PrP ICSM 35 (D-Gen; 1:1,000), anti-PrP 12F10 (Cayman Chemical; 1:200), anti-PrP KG9 (University of Edinburgh; 1:500), anti-phospho-tau (Invitrogen; Thermo Fisher Scientific), AT8, (1:12,00), anti-bA4 (DAKO; 6F3D; 1:50), anti–α-synuclein (Abcam; 4D6; 1: 500), and anti-nonphospho-TDP43 (Abnova; 1:500). Immunostaining was performed on a Ventana Discovery automated immunohistochemical staining platform (Roche), following the manufacturer’s guidelines, using biotinylated secondary antibodies and a horseradish peroxidase–conjugated streptavidin complex and diaminobenzidine as a chromogen. All immunostainings included appropriate positive and negative controls. Slides were digitized at 40-times magnification using a NanoZoomer S360 scanner (Hamamatsu) and representative images for figure preparation were captured using the NZConnect (Hamamatsu) digital slide viewing platform.

## Results

The patient first developed symptoms at age 47 years when his family noted that he was having difficulties organizing his medical appointments. At age 49 years, he had a right-sided parieto-occipital intracerebral hemorrhage, at the time attributed to uncontrolled hypertension. He made a reasonable physical recovery from his stroke but lived at home with a live-in carer from this time. At age 54 years, his cognitive impairment progressed; the live-in carer noted that the patient was disoriented in time and would forget events from earlier in the day. He was referred to speech and language therapy because of language difficulties, who noted in their assessment that he had fluent, grammatically intact spoken language but marked difficulties with repetition and word retrieval.

The patient was reviewed remotely by a cognitive neurologist the following year. Language symptoms were the most prominent difficulty, but there were new navigational difficulties and a dressing apraxia. On examination, the patient’s spoken language was fluent, but there were production errors and reduced use of content words; comprehension was relatively preserved. He had significant difficulty with sentence repetition, managing only words of 2 or 3 syllables. This language syndrome was felt to represent a semantic variant primary progressive aphasia (PPA), although atypical features were noted (phonological errors and impaired repetition, typically associated with logopenic PPA). Memory testing revealed inconsistent retrieval difficulties that were not felt to be typical for a storage issue.

This patient was diagnosed with idiopathic isolated growth hormone deficiency at age 6 years. He received multiple preparations of c-hGH (1974-1985), including Raben and Hartree-Modified Wilhelmi (batches not available for testing). He was diagnosed with secondary hypothyroidism at age 10 years; he was otherwise well as a child. In later adult life, active medical conditions included hypocalcemia, chronic kidney disease, hypercholesterolemia, nonalcoholic steatohepatitis, gallstones, and complex partial seizures.

**Figure 1.**
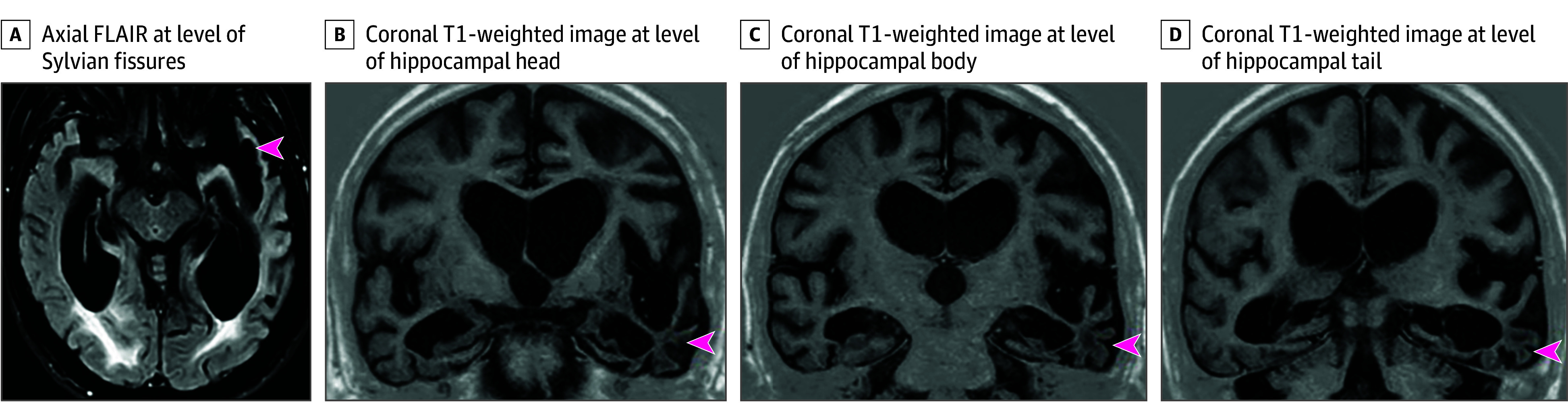
Magnetic Resonance Head Imaging Performed in the Last Year of Life Axial fluid-attenuated inversion recovery (FLAIR) (A) shows asymmetrical widening of the sylvian fissures (red arrowhead), which is more apparent on the left than the right. Coronal T1-weighted images at the level of the head (B), body (C), and tail (D) of the hippocampus show severe mesial temporal lobe volume loss bilaterally, again affecting the left side more than the right (arrowheads).

He was referred to the NPC at age 56 years. By the time of this assessment, the patient was bedbound, fully dependent for personal hygiene and feeding, and doubly incontinent; his spoken language was limited to single words. He had frequent and prominent myoclonic jerks and features suggestive of a swallowing apraxia. Magnetic resonance (MR) head imaging showed progressive atrophy affecting the frontal, parietal, and in particular, temporal lobes with marked hippocampal atrophy, particularly on the left (Figure 1). There were no imaging changes suggestive of CJD. Cerebrospinal fluid (CSF) studies were not performed. Plasma neurofilament light was elevated (58.9 pg/mL; normal range, 0-21.5 pg/mL). Genetic testing for causative variants associated with adult-onset neurodegenerative disorders, including those in *APP*, *PSEN1*, and *PSEN2*, was negative. *APOE* genotype was ε3/ε3; no relevant variants in other risk genes associated with AD (*ABCA7*, *SORL1*, *TREM2*) were identified. Five months after this assessment, the patient died at age 57 years.

**Figure 2.**
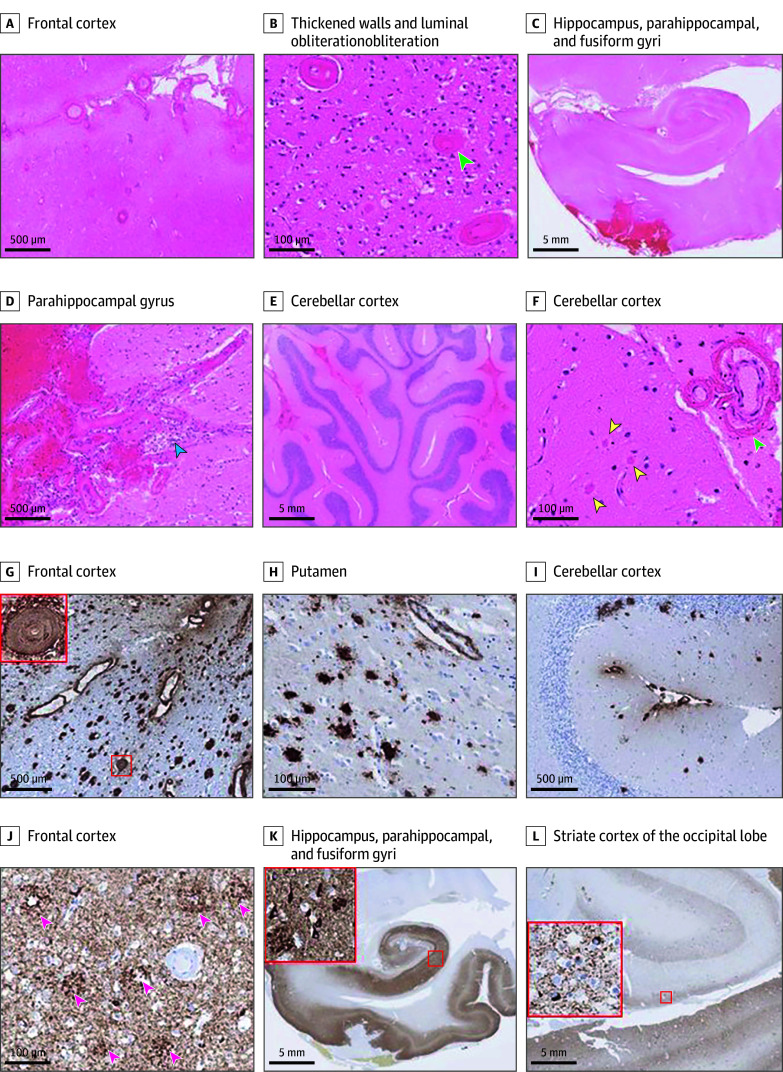
Imaging of Postmortem Findings Hematoxylin-eosin–stained preparations (A-F) show frequent blood vessels in the leptomeninges and frontal cortex, many with prominently thickened hyaline walls (A and B) and near-complete luminal obliteration in some vessels (green arrowhead). Fresh subarachnoid hemorrhage is observed in the parahippocampal gyrus (C and D), accompanied by mild lymphocytic inflammation (blue arrowhead), and also in the cerebellum (E). Leptomeningeal and cortical cerebellar blood vessels have thick hyalinized walls with wall splitting (green arrowhead) and there are brightly eosinophilic deposits in the cerebellar cortex (yellow arrowheads) (F). Immunostaining for amyloid-β (G-I) reveals frequent parenchymal deposits in the frontal cortex (G), putamen (H), and occasionally in the cerebellar cortex (I). There is widespread leptomeningeal and parenchymal cerebral amyloid angiopathy in the cortex (G) with frequent affected blood vessels showing near complete luminal obliteration (inset), occasional involvement in the putamen (H), and frequent leptomeningeal involvement in the cerebellum with occasional amyloid angiopathy in the cerebellar cortex (I). Immunostaining for hyperphosphorylated tau (J-L) shows a dense meshwork of neuropil threads, frequent neurofibrillary tangles, and neuritic plaques across the frontal cortex (J), hippocampus, parahippocampal, and fusiform gyri (K) and also in the striate cortex of the occipital lobe (L). The inset in (K) highlights the neuropil thread, neurofibrillary tangle, and neuritic plaque pathology in the CA1 hippocampal region, and the inset in (L) highlights the neurofibrillary tangle and thread pathology in the striate cortex.

A brain-restricted postmortem examination (Figure 2) revealed bilateral cerebellar tonsillar herniation secondary to a large acute hemorrhage in the left cerebral hemisphere, involving the temporal, parietal, and occipital lobes. There was also widespread subarachnoid hemorrhage over the cerebral and cerebellar surfaces.

Histological examination revealed widespread and severe parenchymal Aβ deposition, characterized by diffuse deposits and plaques with central Aβ cores densely distributed across the neocortex in all lobes, as well as in all hippocampal regions, basal ganglia, midbrain (including the substantia nigra), and cerebellar cortex (Thal phase 5). Cerebral amyloid angiopathy (CAA) was widespread and very severe, affecting vessels in the cerebral and cerebellar leptomeninges, cortex, and putamina. Many cortical vessels across all lobes exhibited wall splitting, and in some, near-complete luminal obliteration by Aβ was observed. However, capillary involvement of Aβ was not seen and there were no features of CAA-related angiitis.

Hyperphosphorylated tau pathology was widespread, manifesting as a dense meshwork of neuropil threads and frequent neurofibrillary tangles in the hippocampi, parahippocampal, and fusiform gyri. Alzheimer-type tau pathology extended across the temporal, frontal, parietal, and occipital lobes, including bilateral involvement of the striate cortex, consistent with Braak and Braak stage VI. Frequent neuritic plaques were present throughout neocortical regions (Consortium to Establish a Registry for Alzheimer’s Disease [CERAD] score 3). The extent and severity of Aβ and tau pathology correspond to the National Institute on Aging and the Alzheimer's Association (NIA-AA) grade A3B3C3, the highest level of AD neuropathological change.

Lewy body pathology was limited to occasional deposits in the amygdala. There was no evidence of misfolded PrP deposition in any brain region or TDP-43 proteinopathy in the limbic or cortical areas. No other specific pathology was identified.

**Figure 3.**
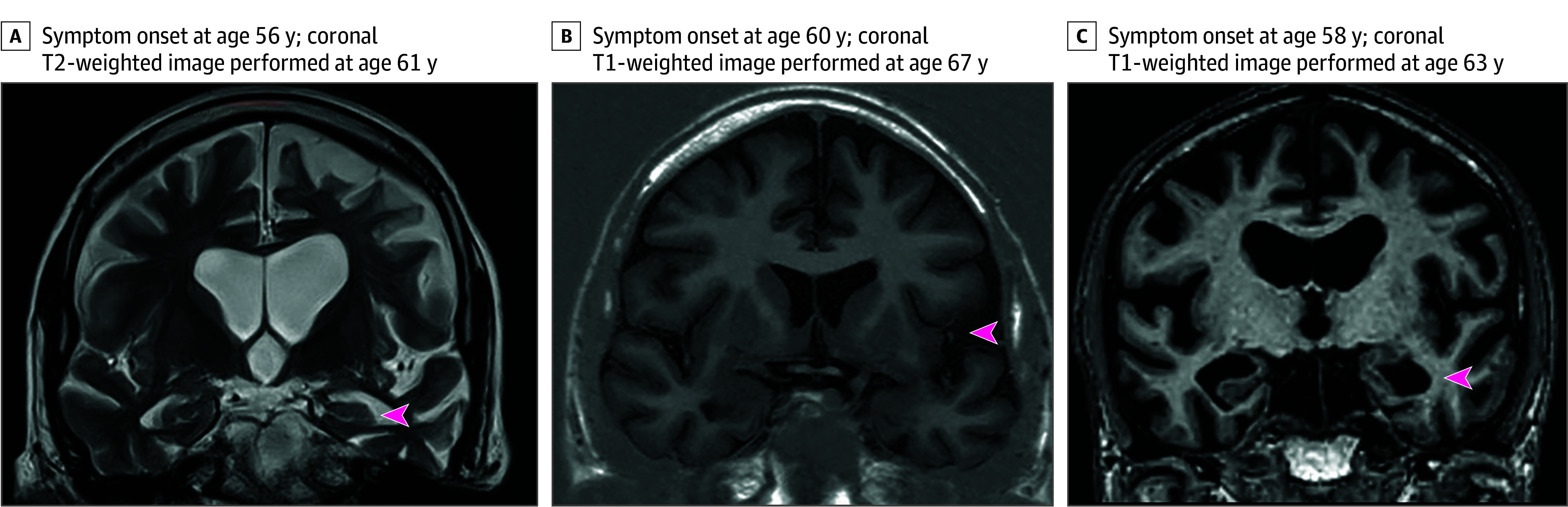
Magnetic Resonance Head Imaging From 3 Cadaveric Pituitary–Derived Human Growth Hormone Recipients With Dementia Syndromes Characterized By Early and Prominent Language Involvement A, Symptom onset at 56 years, imaging performed at 61 years; diagnosed with semantic variant primary progressive aphasia (PPA) prior to our review. Coronal T2-weighted images demonstrate bilateral mesial temporal lobe volume loss with the left more affected than the right (red arrowhead). B, Symptom onset at 60 years and imaging performed at 67 years; diagnosed with semantic variant PPA initially, revised to atypical Alzheimer disease following single-photon emission computed tomography imaging result. Coronal T1-weighted images show widening of the left sylvian fissure (red arrowhead). C, Cognitive symptoms from age 58 years, imaging performed at 63 years; cognitive syndrome felt to be most in keeping with logopenic PPA prior to the review. Coronal T1-weighted images show marked mesial temporal lobe volume loss, more evident on the left (red arrowhead).

In the 12 months since its original report,^8^ the NPC has received referrals for 14 c-hGH recipients; clinical assessments for these patients are ongoing. Notably, in addition to the patient described above, another 3 patients had dementia syndromes with early and prominent language involvement (onset between 56 and 60 years of age). The PPAs observed in these cases were atypical and not easily classified using conventional PPA phenotypes (nonfluent, semantic, logopenic). In 2 of these cases, the diagnosis of semantic variant PPA had been made in life; in 1 case this was revised to atypical AD following single-photon emission computed tomography imaging. The other case was diagnosed with logopenic PPA, with phonemic paraphasias and impaired repetition, in addition to significant apraxia and visuospatial deficits; CSF Aβ42/40 ratio was reduced (0.037; values <0.065 suggest abnormal Aβ deposition). In 2 of these patients, imaging showed asymmetrical (predominantly left) temporal lobe atrophy (Figure 3).

## Discussion

The patient we describe has unequivocal postmortem evidence of AD, with extensive parenchymal Aβ pathology and very severe and widespread cerebral Aβ angiopathy corresponding to the highest level of AD neuropathological change. These findings cannot be attributed to serious childhood illness. Like many of those in our earlier report,^8^ this patient had limited investigations in life, making application of premortem biomarker criteria for AD^13^ impossible. In addition to a dementia, this patient had a lobar intracerebral hemorrhage which, in retrospect, was likely caused by iCAA, highlighting that patients at risk of iatrogenic Aβ pathology can have a mixed clinical presentation.

This patient and 3 others referred to our national service in the last year had cognitive syndromes characterized by prominent and early language involvement; 3 of these patients had been given a diagnosis of semantic variant PPA in life, prior to our involvement, and with asymmetrical temporal lobe atrophy characteristic for this disorder evident on brain imaging in 3 of 4 patients. While semantic variant PPA phenotype is usually associated with TDP-43 pathology, AD pathology is observed in a small proportion of cases (up to 6%).^14,15,16^ Of note in our cases, the language presentation included features unusual for semantic dementia. Our previous report^8^ included another 2 patients with dementia syndromes characterized by prominent language involvement. Language-led dementias (PPAs) are rare, affecting approximately 3 to 4 people per 100 000,^17,18,19^ particularly at younger ages of onset.^20^ Although this observation could indicate that prominent language involvement is an important phenotypic feature of iAD, nonamnestic and other atypical presentations are more frequent in early-onset cases of sporadic AD.^21^ Clinical data from further cases of iAD and careful comparison with equivalent findings in early-onset, sporadic AD will be important for confirming whether this language phenotype is indeed a strain-specific phenomenon specific to an iatrogenic etiology. We also acknowledge that more typical (eg, amnestic) presentations may present to nonneurologists (for example, old-age psychiatrists or geriatricians) or to nonspecialists without being recognized as iAD and without onward referral to our national service. In any case, our observed frequency of language-led dementia in United Kingdom c-hGH recipients (6 patients of approximately 1850 treated individuals, a frequency of around 1 in 300) is well in excess of reported rates for PPA^18^ and early-onset AD.^22^

The extensive tauopathy observed in this case is classical for advanced AD and likely reflects the duration of this patient’s illness (9 years). In our original report,^8^ most patients had shorter symptom duration (range, 2-6 years). One patient had symptoms for 10 years but died without body fluid biomarker testing in life and did not have a postmortem examination; another is still living more than 15 years after symptom onset and had elevation of CSF total tau and phospho-tau measured 11 years after symptom onset. The presence of tau pathology is now recognized in some patients with iCAA (ie, those presenting with intracerebral hemorrhage), with elevation in CSF total tau and phospho-tau,^23,24^ positive tau–positron emission tomography,^25^ and histopathological tau deposition,^26^ all reported in the literature. It is not clear whether tauopathy will eventually develop in all patients with iatrogenic Aβ pathology, should they live long enough, or whether the observed variation in tau pathology also reflects concomitant (and variable) tau seeding at the time of exposure, or the route of exposure (peripheral vs central). Careful longitudinal follow-up will help determine the latency for tau pathology following iatrogenic exposure and confirm the cognitive consequences of this pathology. The extent to which the strain type of iatrogenically inoculated Aβ and/or tau seeds contributes to clinical and pathological phenotype also remains to be established.

### Limitations

As noted by other commentators^27,28,29^ and acknowledged above, there are inherent challenges in proving causality in iAD. In some respects, this resembles early discussions about the causality of variant CJD, now accepted as caused by dietary exposure to bovine spongiform encephalopathy prions.^30^ There are complexities in assessing patients with iAD during life, with personal and practical circumstances limiting comprehensive biomarker and imaging assessments, particularly in severe or late-stage disease. As stated in our earlier report, these findings do not mean that AD is contagious in a conventional sense; transmission has occurred in rare circumstances in the context of cadaveric tissues that are no longer used.

## Conclusions

It is imperative that clinicians remain vigilant for cognitive symptoms in c-hGH recipients and are aware of the emerging evidence for atypical phenotypes associated with iAD; given the age at onset, prominent language, and early dysexecutive features, this syndrome could be mistaken for disorders within the frontotemporal dementia spectrum, highlighting the importance of AD biomarker testing in such patients. Close study of this population provides a unique window into sequencing of, and interactions between, Aβ and tau pathologies, with important implications for sporadic AD and its therapeutic strategies.

## Notes

Supplement. Data Sharing Statement
