# Supplementary material for: High-Level Alzheimer Disease Neuropathological Change Following Iatrogenic Exposure
Source: JAMA Neurol. 2026 Mar 30;83(5):435–41. doi: 10.1001/jamaneurol.2026.0437 (PMC13036638; doi:10.1001/jamaneurol.2026.0437)
Supplement: Supplement. — Data Sharing Statement [file jamaneurol-e260437-s001.pdf]

Data Sharing Statement

Banerjee. High-Level Alzheimer Disease Neuropathological Change Following Iatrogenic Exposure. *JAMA Neurol*. Published March 30, 2026. doi:10.1001/jamaneurol.2026.0437

Data
Data available: No

Additional Information
Explanation for why data not available: Anonymous data can be made available on appropriate request
